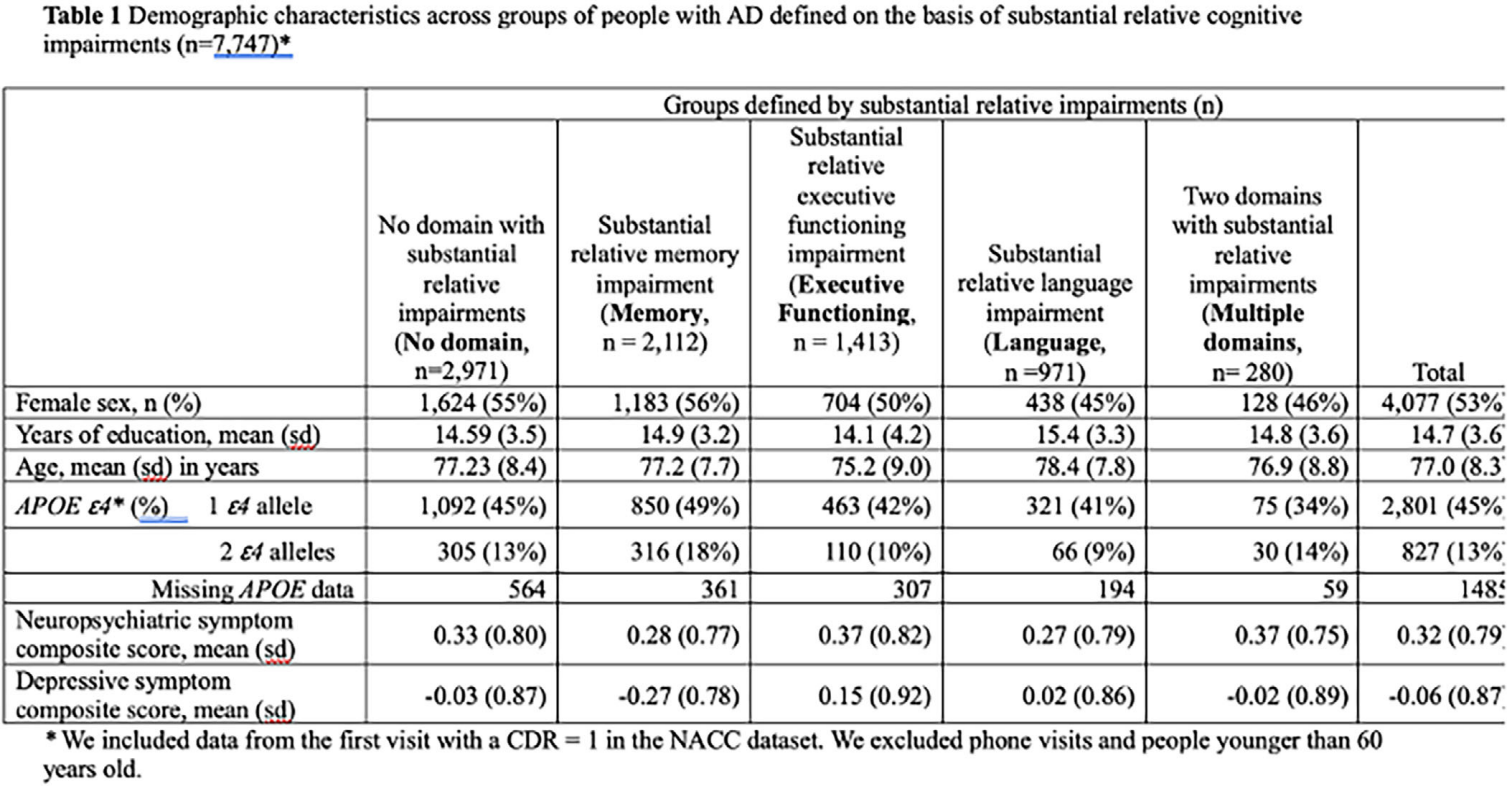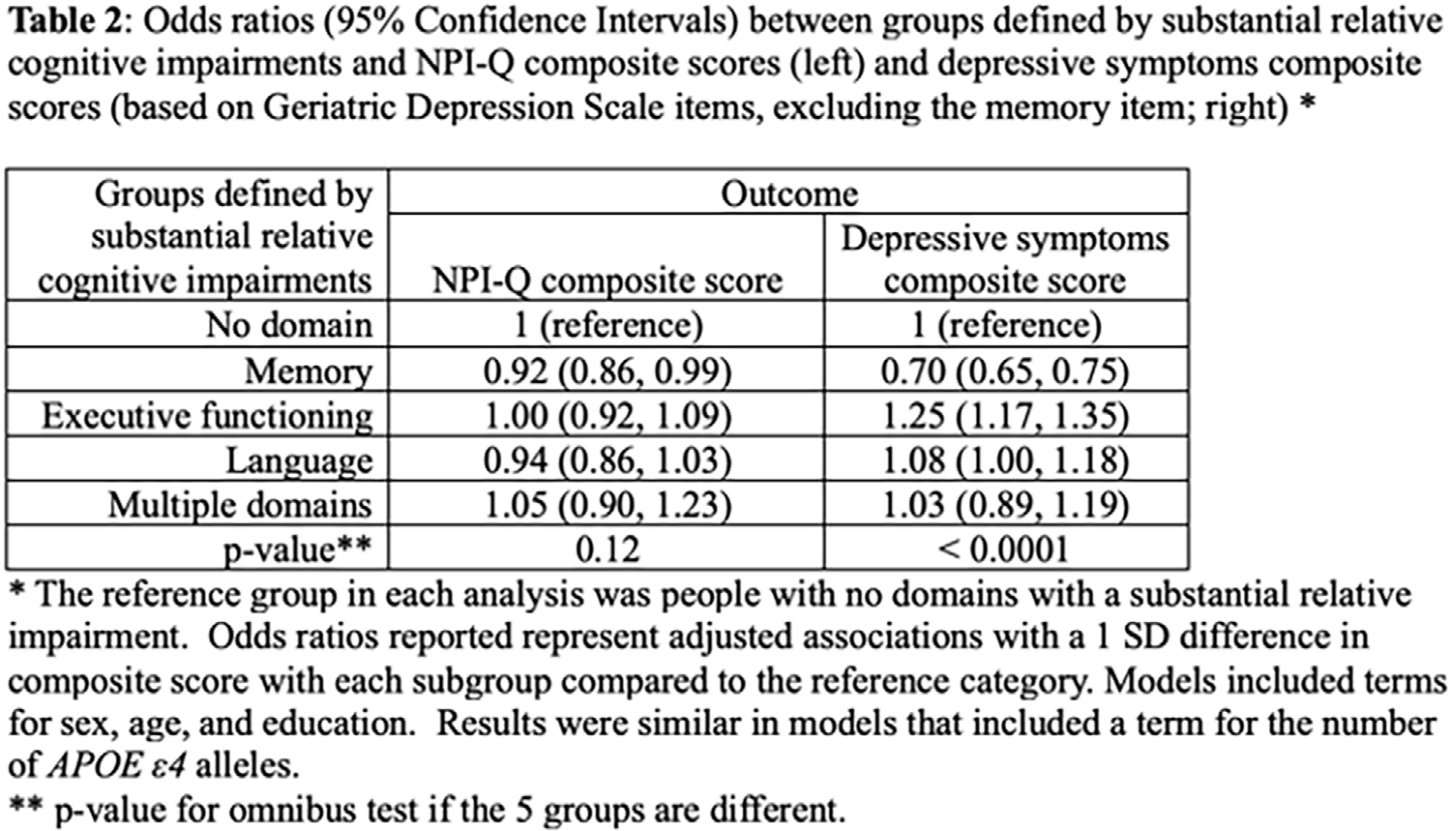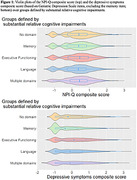# Neuropsychiatric symptoms and depression across cognitively‐defined AD subgroups at the time of AD diagnosis: data from the National Alzheimer’s Coordinating Center (NACC)

**DOI:** 10.1002/alz.093240

**Published:** 2025-01-03

**Authors:** Seo‐Eun Choi, Phoebe Scollard, Shubhabrata Mukherjee, Laura E. Gibbons, Michael L. Lee, Brandon S Klinedinst, Jeanne Gallée, Taiki Sugimoto, Connie Nakano, Walter A. Kukull, Sarah A Biber, Emily H. Trittschuh, Jesse Mez, Andrew J. Saykin, Paul K. Crane

**Affiliations:** ^1^ Department of Medicine, University of Washington School of Medicine, Seattle, WA USA; ^2^ Department of General Internal Medicine, University of Washington School of Medicine, Seattle, WA USA; ^3^ University of Washington, School of Medicine, Seattle, WA USA; ^4^ University of Washington School of Medicine, Seattle, WA USA; ^5^ University of Washington, Seattle, WA USA; ^6^ Department of Epidemiology, School of Public Health, University of Washington, Seattle, WA USA; ^7^ Geriatric Research, Education, and Clinical Center, Veterans Affairs Puget Sound Health Care System, Seattle, WA USA; ^8^ Department of Psychiatry and Behavioral Sciences, University of Washington School of Medicine, Seattle, WA USA; ^9^ Department of Neurology, Boston University Chobanian & Avedisian School of Medicine, Boston, MA USA; ^10^ Indiana Alzheimer’s Disease Research Center, Indianapolis, IN USA; ^11^ Department of Radiology and Imaging Sciences, Center for Neuroimaging, School of Medicine, Indiana University, Indianapolis, IN USA

## Abstract

**Background:**

Neuropsychiatric symptoms are uncommon at Alzheimer’s Disease (AD) dementia diagnosis but are exhibited by nearly everyone during the course of dementia. Depressive symptoms are common in AD dementia. We sought to determine correlations between memory, executive functioning, language, neuropsychiatric symptoms, and depressive symptoms at AD dementia diagnosis, and to characterize neuropsychiatric and depressive symptoms across groups defined by substantial relative cognitive impairments, using data from the National Alzheimer’s Coordinating Center (NACC).

**Method:**

Using confirmatory factor analysis, we derived composite Neuropsychiatric Inventory Questionnaire (NPI‐Q) and Geriatric Depression Scale (excluding the memory question) scores. We defined a reference category of people with AD dementia whose memory, executive functioning, and language scores were similar to each other. Other groups were defined based on relative differences in cognitive domain scores. We used multinomial logistic regression to test if neuropsychiatric and depressive symptoms differed across groups.

**Result:**

Our sample included the diagnosis visit for 7,747 people with AD dementia and CDR = 1 (Table 1). Correlations between cognitive domains ranged from +0.29 to +0.50. Correlations between cognitive domains and NPI‐Q composite scores ranged from ‐0.02 to +0.04 and with depressive symptoms from ‐0.09 to +0.14. The correlation between depressive symptoms and the NPI‐Q composite was +0.17. We did not find a difference in NPI‐Q composite scores across groups, but depressive symptom score differed across groups (p<0.0001), with higher scores in people with relatively greater executive functioning impairments and lower scores in people with relatively greater memory impairments, compared to people with similar domain scores (Figure 1, Table 2).

**Conclusion:**

Composite scores for depressive and neuropsychiatric symptoms facilitate well‐powered investigations of relationships with these clinically salient factors. Neuropsychiatric symptoms had trivial correlations with cognitive domain scores at AD dementia diagnosis, and did not differ across groups defined by substantial relative cognitive impairments. Depressive symptoms were weakly correlated with cognitive domain scores, and differed across groups defined by substantial relative cognitive impairments. More research is warranted to further characterize depression and neuropsychiatric symptoms over the course of AD dementia.